# Understanding the Gastrointestinal Protective Effects of Polyphenols using Foodomics-Based Approaches

**DOI:** 10.3389/fimmu.2021.671150

**Published:** 2021-07-02

**Authors:** Wenwen Zhang, Suzhen Qi, Xiaofeng Xue, Yahya Al Naggar, Liming Wu, Kai Wang

**Affiliations:** ^1^ Institute of Apicultural Research, Chinese Academy of Agricultural Sciences, Beijing, China; ^2^ Zoology Department, Faculty of Science, Tanta University, Tanta, Egypt; ^3^ General Zoology, Institute for Biology, Martin Luther University Halle-Wittenberg, Halle (Saale), Germany

**Keywords:** plant polyphenols, foodomics, polyphenols, gastrointestinal system, gut microbiota

## Abstract

Plant polyphenols are rich sources of natural anti-oxidants and prebiotics. After ingestion, most polyphenols are absorbed in the intestine and interact with the gut microbiota and modulated metabolites produced by bacterial fermentation, such as short-chain fatty acids (SCFAs). Dietary polyphenols immunomodulatory role by regulating intestinal microorganisms, inhibiting the etiology and pathogenesis of various diseases including colon cancer, colorectal cancer, inflammatory bowel disease (IBD) and colitis. Foodomics is a novel high-throughput analysis approach widely applied in food and nutrition studies, incorporating genomics, transcriptomics, proteomics, metabolomics, and integrating multi-omics technologies. In this review, we present an overview of foodomics technologies for identifying active polyphenol components from natural foods, as well as a summary of the gastrointestinal protective effects of polyphenols based on foodomics approaches. Furthermore, we critically assess the limitations in applying foodomics technologies to investigate the protective effect of polyphenols on the gastrointestinal (GI) system. Finally, we outline future directions of foodomics techniques to investigate GI protective effects of polyphenols. Foodomics based on the combination of several analytical platforms and data processing for genomics, transcriptomics, proteomics and metabolomics studies, provides abundant data and a more comprehensive understanding of the interactions between polyphenols and the GI tract at the molecular level. This contribution provides a basis for further exploring the protective mechanisms of polyphenols on the GI system.

## Introduction

Polyphenols are a subclass of phytochemicals, abundantly found in natural products. They are plant-based secondary metabolites that normally contain at least one or more hydroxyl group-linked benzene rings. The chemical structure of polyphenols varies from simple molecules to highly polymerized compounds, including flavonoids, phenolic acids, proanthocyanidins and resveratrol (1). Polyphenols have multiple health benefits owing to their anti-inflammatory (2), anti-oxidant (3), anti-cancer (4), anti-bacterial (5), and anti-diabetic properties (6), inhibition of obesity, and prevention of cardiovascular (7) and cerebrovascular diseases (8). Thus, current scientific research on polyphenols has aroused great interest and significantly attracted the attention of researchers.

The beneficial effects of dietary polyphenols on health depend on their absorption and bioavailability in the body (9). The gastrointestinal (GI) tract is an indispensable digestive organ whose function is crucial to the host’s health, as it regulates the absorption and utilization of nutrients by the body (10). Maintaining good health requires a fully functioning digestive system. A damaged GI system, obstructs GI function, which affects the absorption and utilization of nutrients and thereby threatens host health (11). Gut microbiota are a microbial community inhabiting the GI tract, constituting multiple species that are densely distributed, at approximately 10^14^ microbial cells (12). Gut microbiota are critical to gut health and fulfill multiple tasks in the host (13). They are affected by various internal and external factors, including diet, genetics, and external environmental factors (14).

Most polyphenols found in the natural food matrix are mostly bound and unbound, with the majority of them in the form of bound polyphenols (15), and these polyphenol compounds are considered xenobiotics because of their complex chemical structure, making them difficult to absorb after ingestion (16, 17). Consequently, portions of polyphenols are stored in the large intestine, where they are fermented by the gut microbiota, raising the concentration of short-chain fatty acids (SCFAs) to selectively modulate gut microbiota that can be used by the host (18). Metabolites produced by the catabolism of polyphenols in the intestine may be more bioavailable to gut microbiota and hosts (19). Numerous studies demonstrated that polyphenols maintain gut health by interacting with the gut microbiota (20).

Polyphenols and gut microbiota have interactive effects (21). On the one hand, polyphenols retained in the colon are absorbed and metabolized by gut microbiota and biotransformed into their metabolites, significantly improving bioavailability (22). On the other hand, polyphenols act as a metabolic prebiotics (23), and studies indicate that dietary polyphenols may affect gut microbiota through dual positive effects to benefit GI health, namely, the inhibition of pathogenic microbiota and enrichment of beneficial microbiota (17). When the number of beneficial microbiota in the gut is greater than the number of harmful microbiota, the GI tract’s role and health can be preserved. Therefore, polyphenols ingested at specific concentrations may help maintain GI health by modulating the gut microbiota composition (24). Numerous studies showed that plant polyphenols can alleviate inflammatory bowel disease (IBD) and achieve anti-inflammatory effects by interacting with gut microbiota (19). However, our understanding of the mechanisms by which dietary polyphenols modulate gut microbiota composition and the beneficial effects of polyphenols on the GI tract is severely limited.

**Figure 1:**
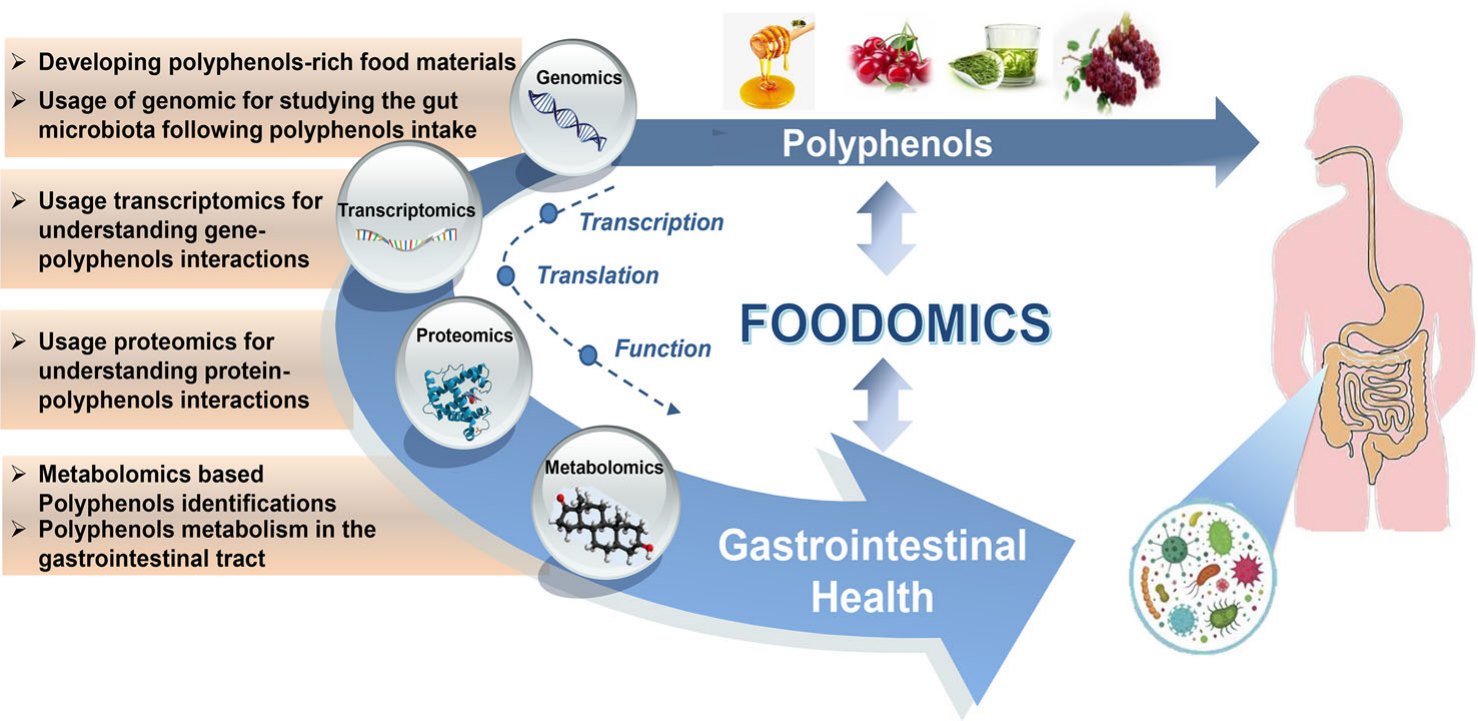
Foodomics helps us understand the interplay between polyphenols, gut microbiota, and host health. Foodomics applies multiple high-throughput omics technologies to provide novel insights into gene transcription, protein expression, and dietary polyphenols metabolism that interact with gut microbiota and host health.

The comprehensive discipline of foodomics was first defined in 2009 by Cifuentes (25) as the study and analysis of the fields of food and nutrition using advanced omics technologies (such as genomics, proteomics, metabolomics, and transcriptomics), to promote consumer trust and health. Integrating these techniques enable researchers to establish links between diet and health. Recent decades have witnessed rapid growth of various applications of foodomics technologies for investigating the beneficial activities of polyphenols on gut health (26). The aim of this review is to further elucidate the growing number of contributions that use the foodomics approach to assess relevant aspects related to the protective effects of polyphenols on GI health, such as: 1) identifying polyphenols and their metabolites after ingestion using GC-MS-and LC-MS/MS-based metabolomics approaches; 2) using genomic/transcriptomic approaches to determine gene expression/transcription and the interactions between genes and polyphenols; 3) exploring protein expression patterns in response to polyphenols in the GI; 4) combining several omics approaches to determine network changes that exist at the cell, tissue, or whole organism level. An overview of integrated foodomics approaches for better understanding the interactions between polyphenols, gut microbiota, and host health is presented in **Figure 1**.

## Polyphenols in the Diet Are Beneficial to GI Health

Dietary polyphenols have been linked to a number of health benefits in the GI tract. Upon reaching the GI tract, part of the dietary polyphenols is absorbed by the intestinal barrier and extensively metabolized in tissues, however, the non-absorbed polyphenols from the small intestine are retained in the colon (27). Most dietary polyphenols are retained in the GI tract as xenobiotics and interact with the microbial community in the intestine during digestion. The variations in polyphenol intake available for absorption and metabolism in the GI tract are dependent on their structures. Polyphenols retained in the GI tract are usually catabolized by gut microbiota (28), and the composition of gut microbiota may also result in differences in the metabolism and bioavailability of polyphenols and their metabolites (29). Polyphenols are metabolized via glucosidase, esterase, dehydroxylation, decarboxylation and demethylation activities by gut microbiota to various derivatives (21), all of which further improve the bioavailability of polyphenols and enhance the benefits to GI health.

Polyphenols play an immunomodulatory role by regulating intestinal microorganisms. The gastrointestinal tract contains a large number of macrophages and neutrophils, which play an immunomodulatory role in the immune system through recognition, uptake, and destruction of intestinal microorganisms (30). The immune system interacts with intestinal microorganisms to maintain the health of the gastrointestinal tract, and polyphenols improve the cellular immune response by regulating these intestinal microorganisms and immune factors, which together maintain a healthy balance in the gastrointestinal tract (31). Indeed, multiple studies have addressed the protective effects of polyphenols on the GI tract (17), dietary polyphenols play a prominent role in altering the gut microbiota, improving barrier function, and inhibiting the etiology and pathogenesis of various diseases, including colon cancer, colorectal cancer (32), IBD, and colitis (33). Polyphenols regulate gut microbiota by anti-microbial activity with bacteriostatic or bactericidal actions and serve as are potential prebiotics enhancing the growth of beneficial microbiota. *In vitro* and *in vivo* studies indicate that cocoa polyphenols supplements stimulate the population of beneficial bacteria, including *Lactobacillus* (34), *Bifidobacterium* (35), *Prevotella*, *Faecalibacterium prausnitzii* (36), *Blautia* (37), *Bacteroides uniformis* (38), and *Lactobacillus reuteri* (39). In turn, cocoa polyphenols supplements inhibited the growth of *Escherichia coli* (*E. coli*) enterohemorrhagic O157: H7, *Salmonella typhimurium*, *Listeria monocytogenes* (34), *Bacteroides*, *Clostridium*, *Staphylococcus* (40), *Lactobacillus-Enterococcus* group (37), and *Clostridium histolyticum* (36). Polyphenols from diverse grapes (mostly phenolic acids, flavonols, favan-3-ols, anthocyanins and hydroxybenzoates) (41) attenuated *Clostridium histolyticum* (42), *Staphylococcus aureus*, *Pseudomonas aeruginosa*, *Klebsiella pneumoniae*, *E. coli*, *Staphylococcus epidermis*, *Enterococcus faecalis*, *Streptococcus pyogenes*, *Haemophilus influenzae*, *Enterococcus casilliflavus*, and *Pneumococcus* (43), and increased the growth of *Lactobacillus-Enterococcus* (42), *Lactobacillus acidophilus*, *Lactobacillus reuteri* (44), *Lactobacillus casei*, *Lactobacillus plantarum* (45).

Colon cancer is one of the world’s most prevalent tumor (46). The inhibition of colon cancer and colorectal cancer may be achieved by polyphenols and their metabolites owing to the inhibition of cancer cell proliferation (47), promoting cancer cell death (48), and regulating intestinal microbes. For instance, the potential of the date palm extract (*Phoenix dactylifera* L.), date polyphenol-rich extract and their metabolites SCFAs in Caco-2 cells was investigated, and both significantly increased the growth of bifidobacteria in human fecal batch cultures to enhance colon health and inhibit colon cancer cell growth (49). Berries are rich in polyphenols, and their inhibitory effects on colorectal cancer progression have been assessed by multiple *in vitro* studies (50). Cranberries polyphenols inhibit colorectal cancer mainly by regulating relevant gene expression, altering cellular signaling pathways, scavenging free radicals in cells, inhibiting cancer cell proliferation and promoting apoptosis (51). Using extracted and purified polyphenols from *P. koraiensis* pinecone (PPP), anti-proliferative activities against colon cancer cells were studied (52). The authors tested different ethanol concentrations (20%, 40% and 60%) in the PPP extract and found the highest phenolic content (57.25 ± 1.83%) in PPP-40 extract that showed the greatest inhibitory effect against LOVO cells. Apoptosis in LOVO cells caused by PPP-40 was mainly mediated through the activation of intrinsic and extrinsic caspase and mitochondria dysfunction (52).

IBD is a global disease characterized by a group of chronic and recurring inflammatory conditions in the GI tract (53). Several *in vivo* or *in vitro* experiments reported that different pathogenesis pathways have been linked to dietary polyphenols which had beneficial effects on the suppression and reduction of IBD symptoms (54). The natural polyphenol resveratrol identified in various plant species, has shown curative effects on IBD *via* the inhibition of NF-κB activation, decreased PGE 2 and PGD 2 levels, inhibition of neutrophil infiltration, as well as reducing COX-2 expression (55). Green tea polyphenols (GTPs) are rich in (-) epicatechin gallate (ECG), (-) epigallocatechin gallate (EGCG), (-) epicatechin (EC) and (-) epigallocatechin (EGC), all of which have a beneficial impact on attenuating IBD. The mechanism of GTP action includes promoted growth of *Bacteroidetes* microbiota, with increased SCFA production and down-regulating the inflammation-relating pathways (56).

## Application of Foodomics and Integration of Omics Tools in Food Sciences

**Figure 2:**
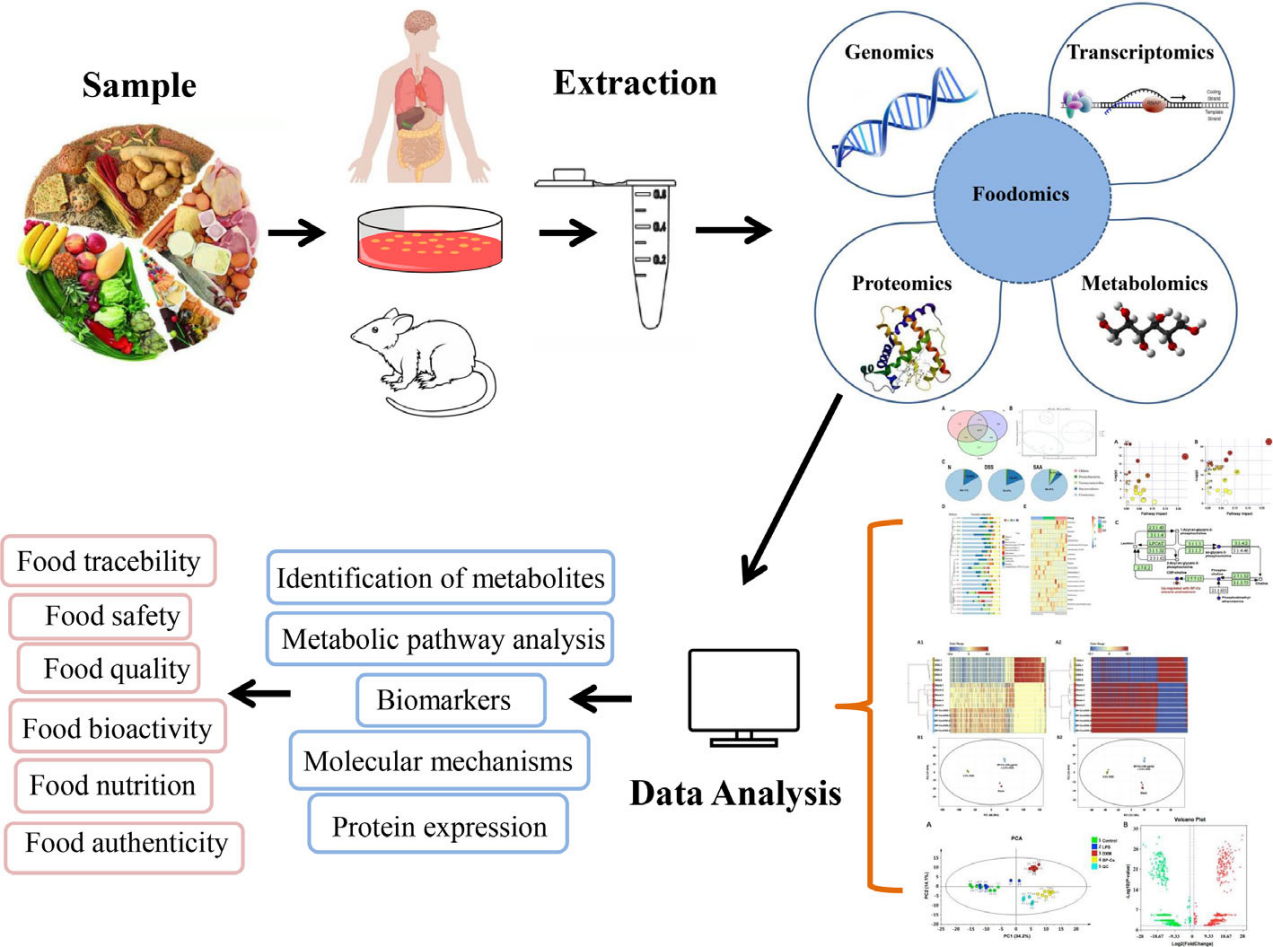
Foodomics-application in food sciences. Research priorities and findings in food science are described intuitively in the form of visual data and graphs using foodomics technology.

Foodomics technologies have advanced rapidly in recent years, which is primarily reflected in existing technologies used in food science, making foodomics methodological research more flexible, as shown in **Figure 2**. Omics technologies mainly include genomics, transcriptomics, proteomics, and metabolomics (57), all of which apply multiple high-throughput omics technologies to investigate related issues in food science. Each omics technology have its own set of research goals, instruments, extraction & separation technologies and data analysis tools. Foodomics helps in the analysis of the biological activities of foods and their potent compounds in order to gain new insights into important molecular mechanisms as well as the exploration and development of novel biomarkers. Foodomics also promotes human health by providing optimal tools to identify information on how dietary nutrition interacts with gene transcription, protein expression, and the metabolism (58). Understanding of foodomics technology is important for researchers to better understand the interactions between polyphenols and gut microbiota.

### Genomics

Genomics refers to the sequencing, assembly and analysis of the structure and function of genomic genes within an organism. The primary goal of genomics is to comprehend the various components of biology and to obtain the maximum amount of genetic sequence knowledge as possible about biological components through experiments and calculations. Among the tools used in genomics, the most powerful and versatile ones are high-density arrays of oligonucleotides or complementary DNAs (cDNAs). DNA arrays are a collection of related DNA spots that represent single genes attached to a solid surface by covalent or electrostatic binding with suitable chemical matrices (59). Another powerful tool for studying the structure and function of the genome is the next-generation sequencing (NGS) technologies. Compared to DNA arrays, NGS technology is capable of processing millions of sequencing reactions simultaneously without requiring a sequence library (often referred to as massively parallel sequencing) (60). NGS enhances the speed of acquisition of DNA sequence information and reduces the sequencing costs (61). Single-molecule sequencing (also called third-generation sequencing systems) is likewise an attractive tool for studying genomics. Single-molecule sequencing, unlike NGS, encounters relatively simple orientation errors, and the sequencing samples are single DNA molecules that cannot be cloned or amplified during the preparation process (62). Single-molecule sequencing allows high-density single molecules expand asynchronously, thereby allowing highly flexible chemical kinetics (63).

Genomics provides opportunities for developing genetically modified crops and livestock with various advantages, including high growth performance, increased yield, disease/pest resistance, and improved nutrient levels in food materials. Genomics techniques enable the determination of the gene compositions of a single plant or farm animal. Based on the constructed “graphic genotype”, plant breeders can find inheritable chromosome sections and accelerate the selection of marker traits, which may reduce the field labor required. Genomic techniques identify beneficial alleles in the genes that govern food properties and select more nutritious and safer crops for better and healthier food (64). Moreover, efforts have been made in using genomics to assess the microorganisms in foods and manage food microbiological issues (65).

### Transcriptomics

The transcriptome is the entire set of RNA transcripts produced by the genome, and it serves as a vital link between gene phenotype and DNA coding information (66). Transcriptomics is the study of all RNA information of a single cell or a group of cells, and it is a paramount tool for understanding the functional elements of the genome and revealing the molecular composition of cells (67). Transcription can be precisely measured through transcriptomics, allowing us to understand the extent and complexity of transcriptomes more comprehensively. Gene expression microarrays and large RNA sequencing (RNA-Seq) are the key methods for analyzing thousands of transcriptomics efficiently and quickly). Gene expression microarray technology was introduced in the 1990s and has since been widely used (68), allowing high-throughput research to advance. In recent years, gene expression microarray technology has been widely applied in the research of emerging diseases, production of new medicines, and the mechanism of food reaction.

The application of microarrays in gene expression are classified into two types according to their nature: microarrays on solid flat or microchip substrates and microarrays on cubic or particle substrates (69). Nevertheless, all samples are first tested to obtain the corresponding signal information, and the signals are subsequently processed with the processing method depending on the platform used. After processing, the relative expression level of each transcript from samples under different experimental conditions is calculated and analyzed to classify differentially expressing genes (70). However, there are several shortcomings in gene microarray analysis because it is based on known sequences, and it is thus impossible to characterize unknown RNA sequences. Consequently, a comprehensive and precise characterization of the transcriptome is impossible (66). RNA-seq however, can supplement this (71) allowing for qualitative and quantitative analysis of any kind of RNA, including microRNAs, messenger RNAs (mRNAs), small interfering RNAs (siRNAs), and long noncoding RNAs. RNA-seq technology can be used for genome-wide high-throughput transcriptomics since it sequences the entire transcriptome (72).

Currently, transcriptomics technologies are widely applied in food crop production. Transcriptomic-based fingerprinting can be used to detect hazardous food constituents or contaminants, including dioxins, xenoestrogens, organochlorine pesticides, mycotoxins phytoestrogens, Maillard reaction products, and estrogen-like chemicals, allowing more effective control of food quality and safety (73). It also assist in elucidating the molecular mechanisms of metabolic transformations and functionalities in food fermentations (66), and demonstrate the effects of dietary nutrients from foods. Moreover, transcriptomics is used to study the alterations of host gene expression due to various dietary interventions. DNA microarray analysis of rats with on an energy restriction diet of 5-30% revealed 72 genes that underwent restriction level-dependent changes (74).

### Proteomics

Proteomics is a complement to genomics and transcriptomics that offers precise biological knowledge for foodomics. The later refers to the use of proteomic techniques to analyze proteins in specific biological food systems on a wide scale. In addition to studying chemical structure and functional proteins, proteomics also investigate proteins alteration effects, quantitative analysis of protein abundance, protein interactions, and their intracellular mechanism exploration (75). Proteomics is dedicated to the qualitative and quantitative analysis of proteins expressed in biological systems at specific times and conditions (76). The procedures of proteomics includes the extraction and separation of proteins, protein digestion into peptides, mass spectrometric (MS) analysis, and then qualitative and quantitative analysis of proteins (77). In generally, there are two methods for protein isolation and separation in proteomics, namely two-dimensional electrophoresis (2-DE); or multi-dimensional liquid chromatography. The 2-DE method of protein isolation and separation is based on the isoelectric point (pI) and molecular mass separation of proteins by 2-DE on polyacrylamide gels, followed by image analysis to classify all discernible spots in the image to provide a reference for subsequent research (78).

The 2-DE process for extracting and separating proteins, on the other hand, has a lot of weaknesses. For example, the performance of the extraction and separation of high-molecular or low-molecular-weight proteins is poor and time-consuming (79). Therefore, multi-dimensional liquid chromatography has been developed, where protein extraction and separation is performed by LC coupled to tandem MS (LC-MS/MS). Currently, the tools used for proteomic analysis to characterize protein samples are MS (80), mainly including matrix-assisted laser desorption/ionization time of flight (MALDI-TOF) and electrospray ion trap (ESI-IT) MS. Both techniques first ionize proteins and then analyze them by MS (81). Proteomics research can be categorized as “bottom-up” or “top-down”. In the “bottom-up” approach, purified protein or complex protein mixtures are first enzymatically digested from the corresponding protein into peptides, and then analyzed using MS (82). The “top-down” approach retains most unstable proteins that were destroyed in the “bottom-up” approach, and performs MS analysis on intact proteins without cleavage (83). Therefore, the “top-down” approach is more stable and reliable than the “bottom-up” approach.

These powerful proteomics methodologies had a major impact on the field of food science. Proteomics was applied in the quality control of various food of biological or transgenic origin using different high-performance separation techniques, combined with high-resolution MS (84). Using 2-DE, the proteomic map of Alfalfa (*Medicago sativa*) was established for the first time, and the protein pattern changes in different processes were studied (85). Proteomics was applied in animal production and health to separate and identify all proteins present in a given tissue or fluid, offering more specific methods for assessing meat maturation, characterizing the proteome changes of post-catch fish muscle, and establishing various production animals proteome maps (86). It is also adopted to identify microbial food contaminants and their toxins (87).

### Metabolomics

The emergence and application of metabolomics is another milestone for foodomics studies. Metabolomics technology focuses on the qualitative and quantitative research of small molecule metabolites (<1,000-1,500 Da), to compare the differences among samples (88). One of the main objectives of metabolomics research is to identify biomarkers, which are molecules that have a direct impact on an organism’s metabolism or metabolic pathways. In general, metabolomics workflows include the following steps: extraction of target metabolites based on research goals; analytical instrument selection and sample preparation; sample on-board testing; collecting data; and using analytical tools for analysis and detection (89). Bioinformatics and chemometrics are analytical tools mainly used for metabolomics data (90).

There are two basic approaches targeted metabolomics and non-targeted metabolomics. Non-targeted metabolomics includes metabolic profiles and metabolic fingerprints (91), while targeted metabolomics is mainly used to analyze key metabolites on specific metabolic pathways, which can be used to investigate the key metabolic alterations caused by specific gene or protein changes. The scope of non-targeted metabolomics is relatively wide, with metabolic fingerprinting focused on comparing changes in metabolite patterns due to changes in internal or external factors (92). Metabolic profiling is focused on studying the differences in related metabolite levels and the effects of corresponding metabolic pathways, which have been applied for the identification of biomarkers in food and for the development of functional food.

The most frequently used data acquisition platforms in metabolomics are nuclear magnetic resonance (NMR), liquid chromatography-MS (LC-MS), gas chromatography-MS (GC-MS), and capillary electrophoresis-MS (CE-MS) (93). Among them, the application of NMR technology was most common in early metabolomics studies. NMR is a powerful analytical technique, for quantifying metabolites and analyzing structural details. It needs small sample size and requires no complex sample preparation procedures such as sample separation or derivatization. However, NMR analysis technology has the limitation of relatively low sensitivity of metabolite detecting (94). MS-based metabolomics technologies have some advantages, and they are mainly used to identify unknown compounds and for quantitative analysis of metabolites (95). The significant advantages of MS analysis technology requiring a small sample volume, high sensitivity, and fast separation speed (96).

LC-MS is the most widely used among MS technologies. It can be applied in the majority of metabolic profiling studies and is a powerful technology that can quantify metabolites and accurately identify the structural information of metabolites. GC-MS technology focuses primarily on the analysis of volatile, non-polar and thermally stable compounds with high separation efficiency and excellent reproducibility, allowing it to analyze complex metabolic mixtures, and it is still extremely useful with the introduction of capillary gas chromatography (97). The appearance and application of CE-MS technology is a further supplement and improvement to LC-MS and GC-MS. CE-MS likewise requires minimal sample volume, and simple sample processing, high separation efficiency, while it exhibits excellent reproducibility and high sensitivity, and can be used to analyze highly polar or charged compounds (92). All these different omics techniques each have their own advantages, and combining several metabolomics analysis technologies will yield complementary analysis results.

Metabolomics technologies have been widely applied in food science. The usage of NMR-based metabolomics in functional food studies aided in the evaluation and characterization of active ingredients as well as the effects of various biomarkers in corresponding diseases (98). Metabolomic-based approaches combining non-targeted and targeted technologies can be applied in food quality testing, detecting chemical contaminants, evaluating food authenticity and assessing food quality (99). MS-based metabolomics were used for food traceability, which accurately determines the basic composition and origin of foods during various processes of manufacturing. Metabolomics may also be used to monitor changes in the metabolomic profiles and identify specific compounds as markers of food degradation (100). It is also used in nutritional epidemiology to identify biomarkers of dietary intake. The earliest biomarker identified using metabolomics after meat intake was trimethylamine N-oxide (TMAO); 1-methylhistidineas was later confirmed as a biomarker of meat consumption (101).

### Integrating Approaches in Foodomics Studies

Foodomics can be used not only for data collection by omic techniques, but also to integrate multiple omic techniques for getting more comprehensive and systematic experimental data (102). Foodomics technologies include chemometrics, epigenomics, bioinformatics and integration approaches. Chemometrics employs mathematical, statistical, and other formal logic-based methods to plan or select optimal measurement procedures and experiments, as well as to analyze chemical data to provide the most important chemical details (103). Chemometrics technology is a subset of metabolomics that involves building a model to define and validate target samples. The methods of identification and classification used in chemometrics technology mainly include unsupervised principal component analysis (PCA), supervised discriminant analysis (DA), hierarchical cluster analysis (HCA), and soft independent modeling of class analog (SIMCA) (104).

The term “epigenetic” simply means “in addition to genetic sequence changes”. The term has evolved to refer to any mechanism that alters gene activity without altering the DNA sequence, resulting in changes that can be passed on to daughter cells (although experiments show that some epigenetic changes can be reversed (105). Epigenetics analysis is a fascinating research area of foodomics that involves analyzing changes in epigenetic status across the entire genome (106). Whole genome research is mainly used to investigate changes in chromosome structure, as chromosome structure can affect gene expression and thus the epigenetic status of the corresponding location (107). Bioinformatics is a technology for reprocessing and analyzing data obtained from various omics technologies. Bioinformatics utilizes a variety of tools to conduct in-depth exploration of data, and to ultimately identify biological significance through functional annotation, genetic and protein data cluster analysis. Biomarkers can be identified and molecular mechanisms can be explained based on bioinformatics, biostatistics and pathway analysis results (108). Integrating multi-omics approaches is necessary due to the unpredictability of the human body and its potential interactions with food. Using multi-omics platform enables researchers to obtain a comprehensive understanding on dietary food components and biological actions in the body.

## Using Foodomics to Clarify GI Protective Mechanisms of Polyphenols

A reliable approach is to integrate foodomics to clarify the GI protective effects of polyphenols with high-throughput molecular technologies that drive it. For genomics, transcriptomics, proteomics and metabolomics studies, foodomics is based on a combination of several analytical platforms and data processing. These omics technologies allow the identification of bioactive compounds of polyphenols and determination of changes induced by polyphenols at the molecular level.

### Identification of Plant Derived Bioactive Polyphenols Using Foodomics

Due to the high complexity of plant polyphenols, the analysis of polyphenols from plant extracts or biological samples has numerous known difficulties in various analytical procedures. Qualitative and quantitative analysis and data processing are two specific bottlenecks of polyphenolics analysis. Foodomics has introduced novel concepts and advanced technologies for identifying bioactive compounds (109). As a result, advanced foodomics technologies were used to improve the analytical methods of polyphenol compounds. We summarized typical examples of using foodomics approaches to analyze the plant derived polyphenols active compounds in fruits (**Table 1-1**), drinks (**Table 1-2**), grain and oil (**Table 1-3**) and other natural products (**Table 1-4**).

**Table 1-1:** MS-based foodomics applied to the determination of polyphenolic active compounds in fruits.

Polyphenol-rich fruits		Counts of Polyphenols	Predominant Polyphenols Identification	MS based tools	References
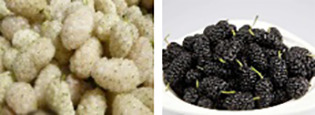	White (*Morus alba L.*) and black (*Morus nigra L.*) mulberry	64	Flavonols; Flavanones; Flavan-3-ols; Flavone; Flavanonol; Dihydrochalcone; Anthocyanins; Hydroxycinnamic derivatives; Phenolics; Hydroxybenzoic acids; Lignans; Organic acids	UHPLC-ESI-MS^n^	(110)
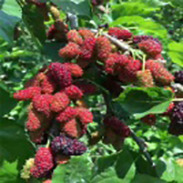	Mulberry cultivars	18	Cyanidin-3-O-glucoside; 3,5-Dicaffeoylquinic acid; Rutin; Quercetin; Quercetin-3-O-glucoside; Cyanidin-3-O-rutinoside; (+)-Catechin; Quercetin-3-O-hexoside; Dihydroxycoumarin; 3-O-Caffeoylquinic acid; Quercetin hexosylhexoside; Quinic acid; Quercetin-O-α-rhamnosyl-triglucoside; Aesculin; Kaempferol hexoside; Kaempferol-3-O-rutinoside; Taxifolin-O-rutinoside; Taxifolin-O-glucoside; Quercetin-3-O-rutinoside-glucoside	HPLC and LC-MS	(111)
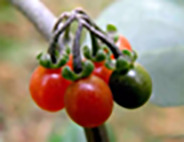	Solanum scabrum and Solanum burbankii berries	18	Delphinidin-3-O-rutinoside-5-O-glucoside; 5-Caffeoylquinic acid;Quercetin-3-O-rutinoside; Quercetin-3-O-glucoside; Petunidin-3-O-rutinoside-5-O-glucoside; 3-Caffeoylquinic acid;Malvidin-3-O-rutinoside-5-O-glucoside; 4-Caffeoylquinic acid; Acetylo-*p*-coumaroylquinic acid; Malonyl-caffeoylquinic acid; Delphinidin-3-O-*p*-coumaroyl-rutinoside-5-O-glucoside; Delphinidin-3-O-feruoyl-rutinoside-5-O-glucoside; Sinapoyl malic acid; Petunidin-3-O-*p*-coumaroyl-hexoside-5-O-hexose; Petunidin-3-O-feruoyl-hexoside-5-O-hexose; Malvidin-3-O-*p*-coumaroyl-hexoside-5-O-hexose; Malvidin-3-O-feruoyl-hexoside-5-O-hexose	UPLC-PDA-Q/TOF-MS	(112)
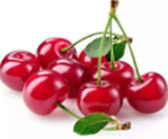	Cherry (*Prunus avium L.*)	9	Hydroxycinnamic acids; Anthocyanins; Flavonoids	LC-ESI-Q-TOF-MS	(113)
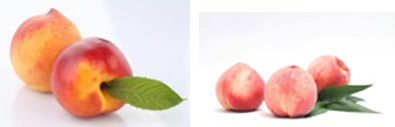	Chinese peach and nectarine	58	Neochlorogenic acid; Catechin; Chlorogenic acid; Protocatechuic acid; Quercitrin; Quercetin; Kaempferol; Hyperoside; Rutin	UPLC-ESI-Q-TOF-MS	(114)
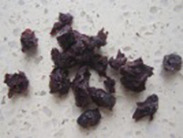	Grape pomace	26	Gallic acid; Syringic acid; Caftaric acid; Cafeic acid; *p*-coumaric acid; Ferulic acid; Polydatin; Piceatannol; trans-Resveratrol; (+)-Catechin; (-)-Gallocatechin; (-)-Epigallocatechin; (-)-Epigallocatechin gallate; Quercetin-3-glucoside; Kaempferol-3-glucoside; Quercetin; OH-tyrosol; Tyrosol	HPLC-MWD and UPLC-ESI-MS	(115)
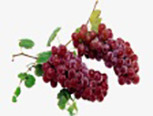	Grape	27	Anthocyanins; Hydroxycinnamic acids; Hydroxybenzoic acids; Dihydrochalcones; Flavanones; Flavonols; Isoflavonoids; Stilbenes	UHPLC-Orbitrap-MS	(116)
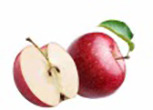	Apple	18	Flavan-3-ols; Flavonols; Dihydrochalcones; Hydroxycinnamic acids	HPLC-DAD-MS^n^	(117)
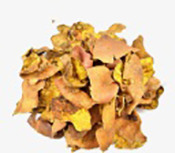	Pomegranate Husk	50	Hydrolysable tannins; Luteolin-3’-O-glucoside; Flavonoids; Hexahydroxydiphenoyl-valoneoyl-glucoside; Galloyl-O-punicalin; Quercimeritrin; Kaempferol-7-O-rhahmano-glucoside; Luteolin-3’-O-arabinoside; Luteolin-4’-O-glucoside	HPLC-QTOF-MS	(118)
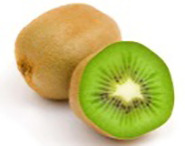	Kiwi fruit	9	Gallic acid; Chlorogenic acid; Catechinic acid; 4-hydroxybenzoic acid; Epicatechin; Rutin; Ferulic acid; Quercetin; Quercitrin	HPLC	(119)
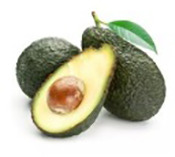	Avocado seeds	17	Luteolin/kaempferol; Catechin/epi-catechin; Quercetin; Caffeoylquinic acid; Luteolin sulfate/kaempferol sulfate; Quercetin sulfate; Kaempferol rhamnoside or isomer; Kempferol hexoside, Luteolin hexoside, Quercetin rhamnoside or isomers; Catechin/epicatechin+ Ph-C3; Kaempferol hexuronic acid or isomer; Quercetin hexoside or isomer; Quercetin hexuronic acid or isomer; Catechin/epicatechin dimers (condensed tannin); Kaempferol disaccharide (hexose-pentose) or isomer; Quercetin disaccharide (hexose-pentose) or isomer; Catechin/epicatechin trimers (condensed tannin)	(-)-ESI-FT-ICR MS HPLC-DAD	(120)

**Table 1-2:** MS-based foodomics applied to the determination of polyphenolic active compounds in grain and oil.

Polyphenol-rich Drinks		Counts of Polyphenols	Predominant Polyphenols Identification	MS based tools	References
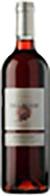	Red wine	43	Procyanidin trimer type B isomer; Gallic acid; Protocatechuic acid-O-hexoside; Gentisic acid; Protocatechuic acid; Caftaric acid; Catechin; Caffeic acid-C-hexoside; Coumaric-O-hexoside; *p*-hydroxybenzoic acid; Caffeic acid; Peonidin-3-O-glucoside; Malvidin-3-O-glucoside; Resveratrol; Cyanidin-O-dihexoside; Quercetin-O-rhamanoside; Epicatechin; Eriodictyol-O-hexoside; Petunidin; Delphinidin-O-dihexoside; Coumaric acid; Myricetin-O-hexoside; Cyanidin; Kaempferol-3-O-rutinoside; Ferulic acid; Piceid acid isomer; Epicatechin-O-gallate; Naringenin; Kaempferol-3-O-glucoside; Quercetin; Quercetin-O-hexoside; Delphinidin; Delphinidin-O-hexoside; Cyanidin-O-hexoside; Petunidin-O-hexoside; Delphinidin acetyl hexoside	HPLC-ESI-LTQ-Orbitrap-MS	(121)
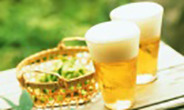	Beer	47	Phenolic acids; Hydroxycinnamoylquinics; Flavanols; Flavonols; Alkylmethoxyphenols; Alpha-and Iso-alpha-acids; Flavones; Hydroxyphenylacetic acids; Prenylflavanoids; Feruloylquinic acid; Caffeic acid-O-hexoside; Coumaric acid-O-hexoside; Sinapic acid-O-hexoside; catechin-O-dihexoside; kaempferol-O-hexoside; apigenin-C-hexoside-pentoside	LC-ESI-LTQ-Orbitrap-MS	(122)
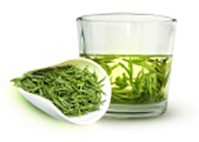	Green tea	86	Phenolic acids; PAs; Flavan-3-ols and their derivatives; Monomeric hydrolyzable tannins; Flavonol and flavonol glycosides; Flavone glycosides	LC-MS	(123)
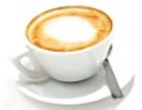	Coffee	11	Gallic acid; DHB; Caffeine; Chlorogenic acid; *p*-Coumaric acid; trans-Ferulic acid; Rutin; Naringin; Resveratrol; Quercetin; Kaempferol	cLC-DAD and LC-MS/MS	(124)
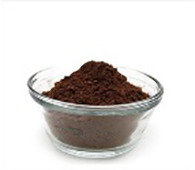	Malaysian cocoa powder	5	Catechin; Epicatechin; Gallic acid; Protocatechehuic acid; Chlorogenic acid	HPLC-UV-ESI-MS/MS	(125)

**Table 1-3:** MS-based foodomics applied to the determination of polyphenolic active compounds in grain and oil.

Polyphenol-rich Grain and Oil		Counts of Polyphenols	Predominant Polyphenols Identification	MS based tools	References
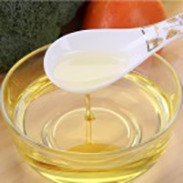	Camellia seed oils	24	Gallic acid; Protocatechuic acid; *p*-Hydroxybenzoic acid; Phthalic acid; *p*-Hydroxyphenylacetic acid; Vanillic acid; Epigallocatechin; Catechin; Caffeic acid; Chlorogenic acid; Epicatechin; *p*-Coumaric acid; Benzoic acid; Epigallocatechin gallate; Ferulic acid; Sinapic acid; Taxifolin; Myricetin; Cinnamic acid; Luteolin; Quercetin; Naringenin; Apigenin; Kaempferol	HPLC-Q-TOF-MS	(126)
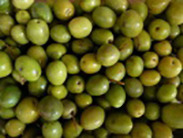	Table olives	16	Hydroxytyrosol tyrosol; Hydroxytyrosol acetate; Salidroside; Catechol; Vanillic acid; *p*-Coumaric acid; Caffeic acid; Verbascoside; Luteolin; Luteolin-7-O-glucoside; Apigenin; Quercetin; Rutin; Oleuropein; (+)-Pinoresinol	LC-ESI-MS/MS	(127)
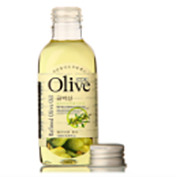	Olive oil	18	Phenolic alcohols; Phenolic acids; Secoiridoids; Flavonoids; Phenolic aldehyde	LC-DAD-ESI-MS/MS	(128)
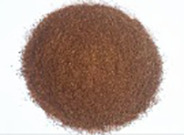	Distiller’s Grains	8	(-)-Epicatechin; Ferulic acid; *p*-Hydroxybenzoic acid; Caffeic acid; Syringic acid; Quercetin; Vanillic acid; Gallic acid	UPLC-MS/MS	(129)
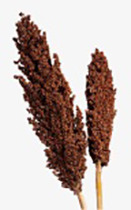	Sorghum Grains	75	Free phenolic acids and derivatives; Flavonoids; Phenylpropane glycerides; Phenolamides	LC-ESI-MS^n^	(130)
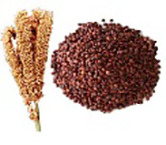	Finger millet	37	Protocatechuic acid; Protocatechuic aldehyde; Catechin; Isomers of catechin-O-dihexoside; Epicatechin; Procyanidin B dimers; Rutin; Quercetin; Apigenin-C-dihexoside; Apigenin-C-pentosyl-C-hexoside; Isomers of apigenin-C-pentosyl-O,C-dihexoside; Quercetin-O-trihexoside; Quercetin-O-trihexoside and procyanidin dimer A; Isomers of quercetin-O-dihexoside; Protocatechuic acid; Protocatechuic aldehyde; Caffeic acid; Sinapic acid; Ferulic acid; *p*-Coumaric acid; *trans*-Ferulic truxilic acid; Ferulic acid dehydrodimers	HPLC-DAD-Q-TOF-MS^2^	(131)
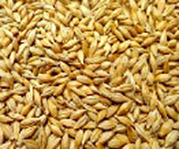	Barley	17	*p*-Coumaric acid isomer; Isoscoparin-2”-O-glucoside; Catechin dihexoside; Catechin-5-O-glucoside; Prodelpinidin B3; Isoorientin-7-O-gentiobioside; Prodelpinidin B; Procyanadin B2; Apigenin 6-C-arabinoside 8-C-glucoside; Catechin; Ferulic acid; Gallic acid; Caffeic acid; *p*-Coumaric; O-Coumaric; Ellagic	Q-TOF LC/MS	(132)
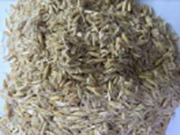	Barley husks	22	Gallic acid; Protocatechuic acid; Catechin-(+); Syringic acid; *p*-Coumaric acid; Hyperoside (quercetin-3-O-galactoside); Naringin; Salviolinic acid; Rutin; 4,5-Di-O-caffeoyquinic acid; Naringenin; Cirsiliol; Apegenin; Acacetin; Beta carotene; Sitosterol; Epicatechin; Caffeic acid; *trans*-Ferulic acid; Silymarin; Stigmasterol; Sitosterol	LC-MS	(133)

**Table 1-4:** MS-based foodomics applied to the determination of polyphenolic active compounds in other natural products.

Polyphenol-rich Other Natural Products		Counts of Polyphenols	Predominant Polyphenols Identification	MS based tools	References
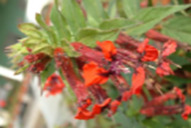	*Cuphea* spp. Leaves	26	Chlorogenic acid; 3-Feruloylquinic acid; 6″-O-Galloylquercimeritrin; Miquelianin; Myricetin 3-O-glucuronide; Myricetin 3-(2″-galloylglucoside); Quercetin; Myricetin 3-glucoside; Myricetin 3′-xyloside; Quercetin 3-(2-galloylglucoside); Kaempferol; Quercetin 3,7-diglucoside; Isoquercetin; Myricitrin; Quercetin-3-sulfate; Kaempferol 3-glucoside; Quercetin-3-arabinoside; Rutin; Kaempferitrin; Kaempferol-galloyl-glucoside; Kaempferol 7-rhamnoside Kaempferol-3-O-rutinoside; Kaempferol-3-xyloside; Kaempferol-3-glucuronide; Quercetin-acetyl-glucuronide;	UHPLC using ESI-Q-TOF	(134)
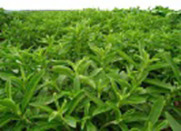	Stevia rebaudiana leaves	55	Phenol diglycoside; Caffeoyl quinic acids; Caffeoyl shikimoyl hexose isomers; Caffeoyl shikimic acids; Feruloyl quinic acid; Dicaffeoyl quinic acid isomers; Kaempferol caffeoyl rutinoside Flavonols glycosides; Kaempferol-3-O-hexoside; Quercetin caffeoyl rutinoside; Quercetin dimethylether-3-O-hexoside; Quercetin3-O-deoxyhexoside; Apigenin7-O-hexoside; Kaempferol-3-O-pentosyl deoxyhexoside; Dicaffeoyl quinic acid isomer; Kaempferol 7-O-deoxyhexoside	UHPLC-ESI-QqTOF-MS/MS	(135).
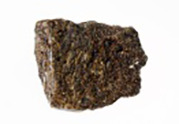	Chinese propolis	11	Gallic acid; Chlorogenic acid; Caffeic acid; (+)-Catechin; *p*-Coumaric acid; (−)-Epicatechin; Taxifolin; Myricetin; Luteolin; Quercetin; Ferulicacid	LC-MS/MS	(136)
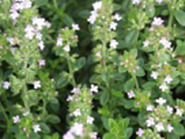	Thymus x citriodorus	10	Eriodictyol-di-O-hexoside; 5′-Hydroxyjasmonic acid 5′-O-hexoside; Eriodictyol-O-hexoside; Quercetagetin dimethyl ether O-hexoside; Eriodictyol-O-hexoside; Luteolin-5-β-O-glucoside; Naringenin-O-hexoside; Eriodictyol-O-hexuronide; Luteolin-7-α-O-glucuronide; Luteolin-7-O-glucoside; Chrysoeriol-7-β-O-glucoside; Apigenin-7-β-O-glucuronide; Rosmarinic acid; 3′-O-(8″-Z-Caffeoyl) rosmarinic acid	HPLC-ESI-MS/MS^n^ NMR	(137)
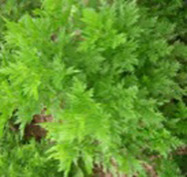	Folium Artemisiae Argyi	38	Hydroxybenzoic acids; Hydroxycinnamic acids; Flavonoids; Methoxylated flavones	UHPLC-Q-Orbitrap-MS/MS	(138)
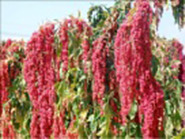	Djulis (*Chenopodium formosanum Koidz.*)	22	Vanillic acid; Quercetin-acetyl-rutinoside; Quinic acid; Hydroxyphenylacetic acid; Caffeoyl-putrescine; Hydroxyphenylacetic acid pentoside; Vanillic acid hexoside; Quercetin-acetyl-rutinoside hexoside; Rutin; Rutin-O-pentoside;Quercetin-3-O-(coumaroyl)-rutinoside; Quercetin-3-O-(coumaroyl)-rutinoside pentoside; Quercetin-3-O-(coumaroyl)-rutinoside deoxyhexoside; Quercetin-acetyl-rutinoside hexoside Glucuronide; Caffeoyl-spermine-conjugate; Querctin-acetyl-glycoside	HPLC-DAD-ESI-MS/MS	(139)
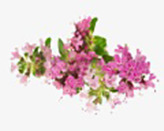	Thymus schimperi Ronniger	23	Eriodictyol; Luteolin-7-O-glucoside; (Epi) gallocatechin; Luteolin-7-O-glucoronide; Luteolin-4′-O-(rhamnosyl) glucoside; Luteolin-6-C-pentoside-8-C-hexoside; Luteolin-6-C-glucoside; Chryseoriol-7-O-glucoside; Apigenin-7-O-(acetyl-apiosyl) glucoside; Luteolin-7-O-(2′′-apiosyl-acetyl)glucoside; Luteolin-6-C-pentoside; Salvianolic acid A; Dihydroxytrimethoxy flavone; Luteolin-7-O-(acetyl-apiosyl) xyloside; Luteolin-7-O-(dipentosyl) glucuronide; Luteolin-7-O-glucuronide-3′-O-glucoside; Luteolin; Trihydroxy-dimethoxyflavone; Hydroxy-tetramethoxyflavone; Hydroxy-trimethoxyflavone; Hydroxy-trimethoxyflavone isomer; Trihydroxy-trimethoxyflavone; Hispidulin	HPLC-ESI-MS/MS	(140)

For the application of foodomics technology in understanding the GI protective effects of polyphenols, we provides an overview of recent studies, on polyphenols or polyphenol-rich foods, the foodomics technology applied (genomics in **Table 2-1**, transcriptomics and protemics in **Table 2-2**, metabolomics and multi-omics in **Table 2-3**), experimental model and dosage, and major findings.

**Table 2-1:** Genomics in understanding the GI protective effects by polyphenols and polyphenolic-rich foods.

Polyphenolic-rich foods		Technique	Experimental model and dosage	Major findings	References
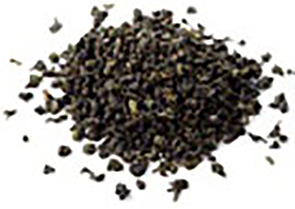	Oolong tea	16S sequencing Next generation sequencing	Six-month-old SD rats	The phylum Bacteroidetes was increased in response to GTP in a dose-dependent manner, the consistent supply of GTP to the gut microbial ecosystem could increasing the abundance of beneficial species and improve the microbial functions, decrease the potential pathogenic species	(141)
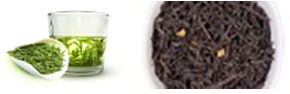	Green and Dark Tea	16S rRNA sequencing	Colitis mice by a fecal microbiota transplantation	GTE and DTE ameliorate chemical induced-colitis by modulating gut microbiota	(142)
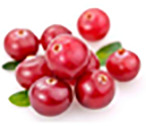	Cranberry	16S rRNA sequences	High fat/high sucrose fed C57BL/6J mice	Lower intestinal triglyceride content and to alleviate intestinal inflammation and oxidative stress	(143)
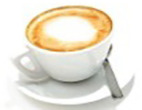	Coffee	16S rRNA gene-based real-time	Diet-induced obese CD rat	Coffee consumption increase in Firmicutes-to-Bacteroidetes ratio and Clostridium Cluster XI, resulted in augmented levels of Enterobacteria	(144)
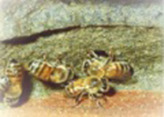	Green propolis	16S rRNA sequencing	C57BL/6 mice	High-fat diet promoted an increase in Firmicutes without a significant decrease in Bacteroidetes	(145)
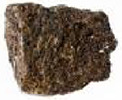	Propolis	16S rRNA sequencing	Male SD rats	0.3% propolis supplementation had a significant increase in gut microbial diversity including Proteobacteria and Acidobacteriaphyla	(146)
(-)-Epigallocatechin 3-O-(3-O-methyl) gallate		16S rRNA sequencing	Human HFD-induced obesity mouse	Enrichment of Bacteroidetes and genes	(147)

**Table 2-2:** Transcriptomics and Proteomics in understanding the GI protective effects by polyphenols and polyphenolic-rich foods.

Polyphenolic-rich foods		Technique	Experimental model and dosage	Major findings	References
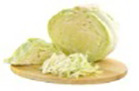	Plums and cabbages	2D-DIGE	Cellular models (Caco-2, Caco-2/HT-29-MTX, and THP-1) of the intestinal epithelium	Different model result in different strengths of response, the Kale digesta demonstrated a high impact on different important antioxidant enzymes	(148)
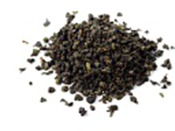	Oolong tea	Microarrays	Multidrug resistance targeted mutation (Mdr1a-/-) mice	GrTP can ameliorate inflammation in the colon of the Mdr1a-/- mouse model of IBD	(110)
		Two-dimensional gel electrophoresis			
		LC-MS			
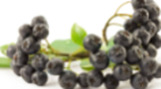	Chokeberry juice	Gene expression microarray analyses	Human model of colon cancer Caco-2 cells	Exposure of Caco-2 cells to pre-digested chokeberry juice resulted in inhibition of both cell proliferation and viability	(149)
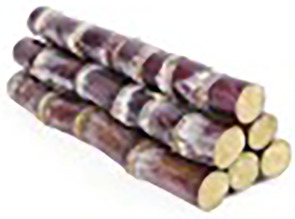	Sugarcane	LC-MS/MS	LPS-stimulated SW480 colon cancer cells	up regulation of the oxidative stress mediator SELH, suppress the phosphorylation of NFκB and inhibit secretion of the pro-inflammatory cytokine IL-8. and contributes to the regulation of important signaling proteins including PKA, PKCβ, c-Jun, EGFR and SIRT1	(150)
Quercetin		MALDI-FT-MS	Male inbred F344 rats	The changes evoked by quercetin can inhibit colorectal cancer	(151)
		MALDI-TOF/TOF-MS			

**Table 2-3:** Metabolomics and multi-omics in understanding the GI protective effects by polyphenols and polyphenolic-rich foods.

	Polyphenolic-rich foods	Technique	Experimental model and dosage	Major findings	References
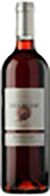 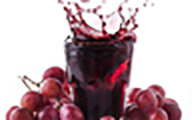	Red wine and grape juice	^1^H-NMR	31 men and 22 women; mean SD age 57.6 ± 1.3 years; mildly hypertensive; non-smokers	The mixture of grape juice and wine extract induced a reduction in isobutyrate, indicate that polyphenols are able to modulate the microbial ecology of the gut	(152)
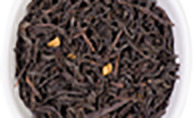	Black tea extract and red wine/grape juice extract	^1^H-NMR	Five-stage *in vitro* GI model	BTE and RWGE modulate microbial SCFA production	(153)
		GC-MS			
		LC-MS			
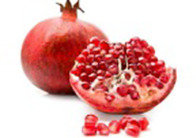	Pomegranate	UPLC-ESI-QTOF-MS/MS	Colorectal cancer (CRC) patients	High punicalagin content hampered urolithins formation	(154)
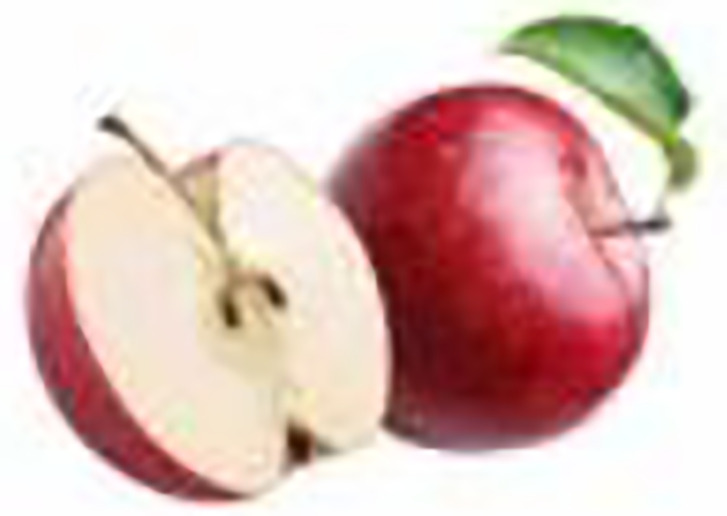	Apple	16S rRNA gene sequencing	SD rats	127 proteins were differentially expressed and resulted in 123 fecal metabolites; there was a strong negative linear relationship between the relative abundance Firmicutes and Bacteroidetes in the high-fat group	(155)
		Mass spectrometry			
		Gas chromatography time-of-flight mass spectrometry			
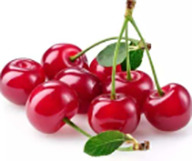	Tart cherries	16S rRNA gene sequencing LC/MS	In vitro incubations were performed by mimicking gastric, intestine and colon conditions	Resulted in large increases in Bacteroides and Collinsella, moderate increases of specific Firmicutes, Enterobacteriaceae and Bilophila	(156)
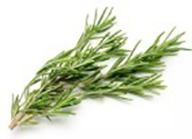	Rosemary	Microarray analysis 2-DE	Human HT29 colon cancer cell	Rosemary polyphenols against colon cancer cells	(157)
		MALDI-TOF/TOF-MS			
		CE-MS			
		UPLC-Q/TOF-MS			
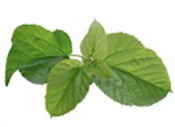	Mulberry Leaf	UPLC Triple TOF MS/MS	SD rats	Gut Environment is Altered by Mulberry Leaf	(158)
		GC-MS			
Dietary polyphenols		CE-TOF MS	Human HT29 colon cancer cells	Reduced glutathione/oxidized glutathione ratio and significant alterations in polyamines content	(26)
		RP/UPLC-TOF MS			
		HILIC/UPLC-TOF MS			

### Gene-Based Genomics and Transcriptomics to Investigate GI Protective Mechanisms of Polyphenols

Foodomics technologies accurately analyze polyphenol compounds in both qualitative and quantitative aspects. Moreover, gene-based genomics and transcriptomics can be used to study the interaction between polyphenols and the GI tract. Owing to continuous optimization of gene-level research technology, gene expression microarray technology has developed as a crucial analytical technology in the transcriptomics field to investigate the interactions between genes following intake of bioactive constituents from food (159). Alberto et al. applied gene expression microarray technology to investigate the effect of polyphenols from enriched extract of rosemary on two colon adenocarcinoma cell lines. Differences in the G2/M arrest inhibition were found in the two colon adenocarcinoma cell lines after treatment with an enriched extract of rosemary. Rosemary polyphenols induced a low degree of apoptosis in colon adenocarcinoma cell lines and the results also indicated multiple other signaling mechanisms that may lead to apoptosis of colon cancer cells (160).

Dolara et al. (141) used transcriptomics to investigate 5,707 expressed genes to further determine the molecular effects of wine polyphenols on colonic mucosa in F344 rats. Wine polyphenols may protect the colonic mucosa by improving intestinal function and having anti-colonic cancer activities by reducing oxidative damage, modulating the colonic microbiota and down-regulating the expression of genes involved in metabolism, transport, signal transduction and intercellular signaling. Wang et al. (161) evaluated the interaction of green tea polyphenols (GTPs) and gut microbiota through transcriptomics. In that study, Female Sprague-Dawley (SD) rats were treated with GTP for 6 months. 16S ribosomal RNA (rRNA) amplicon sequencing (16S-seq) and Shotgun metagenomic community sequencing (SMC-seq) were then used to determine the effect of GTP on the intestine microbiota and the possible connection between improvements in and the beneficial effects of GTP. The SD rats that were treated with GTP over long time periods exhibited a dose-dependent modification of *Bacteroides* and genes related to energy metabolism, which proved to be beneficial for weight control and maintenance. Yang et al. (162) studied the inhibitory effect of combined polyphenols on colitis-related carcinogenesis (CRC) in mice by the 16S rRNA gene sequence. The data show that the gut microbiota plays a key role in the treatment of CRC, bound polyphenol of the inner shell treated altering the diversity and overall structure of the microbiota in tumor-bearing mice, and also exerts a regulatory effect on 17 signaling pathways involved in related genes.

### Protein-Based Proteomics to Investigate GI Protective Mechanisms of Polyphenols

Proteomics is employed to further the understanding of the relationship between the GI tract and polyphenols at the protein level. It assesses the functional protein changes of probiotics in the gastrointestinal transit, metabolic processes using different protein identification approaches by studying the beneficial effects of probiotics on the gastrointestinal tract and the mechanism of action (163). Proteomics is mainly based on MS technology, and it focuses on obtaining functional information about the interaction of polyphenols with the GI tract and related pathway by assessing protein expression changes. Valdés et al. (164) used a proteomics strategy to explore the effect of polyphenol-rich rosemary extract at various concentrations on HT-29 human colon cancer cells for 2, 6 and 24 hours. Nano-liquid chromatography-tandem mass spectrometry (nano-LC-MS/MS) was combined with stable isotope dimethyl labeling (DML) technology to quantitatively examine relative changes in the protein. Rosemary extract, which is rich in polyphenols protected intestinal health with anti-proliferative effects by inducing proteomic changes in cells of HT-29 colon cancer, reducing aggregates formation and stimulating autophagy. Barnett et al. (165) used transcriptomics and proteomics to assess the effect of dietary intake of green tea extract rich in polyphenols (GrTP) on human colon IBD. They used 2-DE and LC-MS technologies to investigate gene expression in the colon and protein expression changes in the Mdr1a ^−/−^ mouse as an *in vivo* model of humans. GrTP reduced intestinal inflammation in the colon of the mouse model of IBD. Li et al. (166) investigated the inhibitory effect and anti-bacterial activity of catechin on *Escherichia coli* O157:H7 cell lines *in vitro* and in simulated human gastrointestinal environment by proteomics. The changes in protein expression were studied by 2-DE that showed changes in the expression of 34 proteins in the bacterial proteome, of which 2 were up-regulated, 12 were down-regulated and 20 were lost. It was shown that catechin had an inhibitory effect on EHEC O157:H7, and the specific mechanism of action must be studied in conjunction with *in vivo* studies.

### Metabolomics Based on Microbial/Colonic Metabolic Metabolites to Investigate GI Protective Mechanisms of Polyphenols

Several researchers have highlighted metabolomics as an important future direction of foodomics, owing to its ability to characterize related biological functions and phenotypes (167). Metabolomics are likewise a suitable omics technology to target the metabolic pathway and understand molecular mechanisms of the metabolism. Metabolomics technology has been used in studies investigating the interaction of polyphenols with the GI tract through changes in the detection of small molecule metabolites. Fernández-Arroyo et al. (168) used nano LC-ESI-TOF-MS to study the anti-proliferative effects of phenolic compounds extracted from extra-virgin olive oil in treating SW480 and HT29 human colon cancer cell line. The analysis of cytosol and cytoplasmic metabolites revealed the presence of numerous phenolic compounds and their metabolites, mainly quercetin and oleuropein aglycones (and their derivatives), in SW480 and HT29 cell lines, which in turn affect cell signaling pathways and lead to apoptosis. Li et al. (169) used UPLC-Q-TOF/MS metabolomics to investigate *in vitro* GI defensive effects of polyphenol-rich bee pollen (BP) extracts on the dysfunction of the Caco-2 intestinal barrier induced by dextran sulfate sodium (DSS). Throughout the early stages of DSS-induced colitis, findings revealed that BP had significant therapeutic ability. The metabolomic results indicate that BP and DSS-treated Caco-2 cell metabolites are significantly different compared with the blank-treated group, and the metabolic pathways involved had the largest effect on the glycerophospholipid metabolic pathway, indicating that BP treatment suppressed the inflammatory response by regulating the cells’ own metabolites and metabolic pathways.

Di Nunzio et al. (170) combined proteomics and transcriptomics technologies to investigate the changes in the distal colon mucosa of F344 rats evoked by dietary quercetin. Dietary quercetin in the distal colon mucosa had an inhibitory effect on colorectal cancer by enhancing expression of tumor suppressor genes, cell cycle inhibitors, genes involved in xenobiotic metabolism and enhancing the inhibitory effect on the MAPK pathway. To investigate the anti-proliferative effects of dietary polyphenols on HT-29 human colon cancer cells, Ibánez et al. (171) developed a multiplatform analysis that combined CE, RP/UPLC, and HILIC/UPLC, both coupled to TOF-MS for metabolomics analysis. Their findings revealed that dietary polyphenol treatment altered 22 closely related metabolites in HT-29 cells, which has the potential to for inhibit colon cancer.

### Multiple Omics Platforms for Understanding the Protective Effects of Polyphenols Against GI Disorders

With the technological development of foodomics, food science studies based on data generated from sequencing approaches and combining two or more technologies (“multi-omics”) are considered as more reliable, which also allows for integrating system-level approaches. The multi-omics approach has a more comprehensive and systematic analysis capability, which is more conducive for in-depth investigation of complex issues in food science. Dietary polyphenol interventions for GI disorders involve multiple molecular and biochemical mechanisms during the process of biotransformation and absorption. Integrated multi-omics analysis is necessary to obtain comprehensive omics-data to identify the genes, proteins and metabolites involved in metabolic regulation, and further construct metabolic pathways to comprehensively analyze the mechanism of polyphenols for gastrointestinal protection, and further elucidate the complex network of interactions between the dietary polyphenol, GI tract, and host (156). Mayta-Apaza et al. confirmed that polyphenol-rich tart cherries regulate intestinal health by increasing the quantity of beneficial microbiota in the human colon. To determine how gut microbiota were influenced by polyphenol metabolites from polyphenol-rich tart cherries in the human colon, they authors conducted bacterial fermentation assays on polyphenol-rich tart cherry concentrate juices and pure polyphenols (and apricots) *in vitro* and assessed the results based on 16S rRNA gene sequence and metabolomics. *In vitro*, gut microbiota metabolized polyphenols into 4-hydroxyphenylpropionic acids and tart cherries modulate the increase of *Bacteroides*. *In vivo* data showed decreased *Bifidobacterium*, *Bacteroides*, and increased levels of *Collinsella*, *Lachnospiraceae*, *Ruminococcus* in individuals with high *Bacteroides*. Low *Bacteroides* individuals had decreased *Collinsella*, *Lachnospiraceae*, *Ruminococcus*, while *Bacteroides*, *Prevotella*, *Bifidobacterium* levels increased (157).

Ibánez et al. applied transcriptomics, proteomics, and metabolomics on mutiple platforms (microarray analysis, MALDI-TOF/TOF-MS and CE/LC-MS) to examine the anti-proliferation effect of polyphenols extracted from rosemary on the total gene, protein and metabolite expression in human HT29 colon cancer cells. The results demonstrated that the dietary polyphenols from rosemary are effective in inhibiting HT29 colon cancer cell growth and proliferation (172). Zhou et al. investigated the effect of GTPs on the metabolic regulation of gut microbiota in SD rats, and analyzed the key metabolites in rat intestinal contents by GC-MS metabolomics and HPLC metabolic methods. They found that GTPs treatment reduced the level of caloric carbohydrates and regulated the metabolism of bile acids, fatty acids and amino acids metabolized by gut microbiota (173). To examine the metabolites present in the gut microbiota-dependent mitochondrial tricarboxylic acid (TCA) cycle and urea cycle of GTPs, 16S rRNA gene sequencing and hydrophilic interaction liquid chromatography (HILIC)-heated electrospray ionization (HESI)-tandem liquid chromatogram mass spectrometry (LC-MS) were used (174). They found that GTPs enhanced the energy conversion and maintained gut health by increasing the mitochondrial TCA cycle and intestinal microbiota urea cycle in rats.

## Conclusions and Future Perspectives

Polyphenols are particularly complex and ubiquitous components of our daily foods sourced from plants, vegetables and fruits. It has shown potential for prevention and treatment of GI disorders, and the interactions between polyphenols and gut microbiota have gained significant attention due to their relevance to bioavailability and host health. Latest advancement of innovations in foodomics have significantly accelerated our understanding of food science and allowed a more comprehensive understanding at the molecular level of the interactions between polyphenols and the GI tract. However, while awareness of the polyphenol-GI tract relationship is growing, there is still a long way to go. There are evident shortcomings and limitations of the interactions between polyphenols and the GI tract. The majority of polyphenols in the natural food matrix are in the form of bound polyphenols that cannot be directly metabolized. As xenobiotics in the GI tract, polyphenols must be metabolized and transformed by the gut microbiota in the colon before they can function for the hosts. Numerous studies we reviewed which based on *in vitro* experiments, used polyphenol extracts from natural foods or single polyphenol compounds to directly affect intestinal cells using foodomics approaches, and the results obtained may be different from the actual state *in vivo*.

In fact, the digestion process of polyphenols in the colon is highly complex, and gut microbiota play a very important role in the metabolism of polyphenols. Most of the digestion and metabolism of polyphenols is done through gut microbiota. Meanwhile, polyphenols also play a beneficial role in regulating the composition of gut microbiota, even if the results of *in vitro* experiments to explain the protective effect of polyphenols on the GI tract or the metabolism of polyphenols in the GI tract are not sufficient. Animal experiments or simulated GI experiments will reduce the variability in experimental results for further understanding of the GI protective effects of polyphenols. Integrating foodomics to elucidate the GI protective effects of polyphenols also has several limitations. Notably, polyphenols are a generic term for a class of compounds, and the polyphenols in different natural foods vary significantly. Not all polyphenols in natural foods have common beneficial biological activities, and natural foods may exert their biological activities through one or more polyphenol compounds. Thus further research on the biological activities of polyphenol monomers in natural foods is required to explore and understand the bioactive compounds in natural foods. For instance, foodomics technologies result in massive amounts of data, which require extensive bioinformatics analysis. The comparison with other studies is challenging, due to the lack of information at the molecular level of all cellular processes, which indicates that there are gaps in showing effectiveness. Coincidentally with the applications and continuous development of high throughput technologies, there is a demand to develop more comprehensive tools to shorten the data processing time. Despite current studies using combined analysis methods, there is a lack of comprehensive data at the molecular level. In the future, studies at multiple molecular levels of genes, proteins, and metabolites are required to comprehensively understand the GI protective effects of polyphenols.

## Author Contributions

WZ and SQ: Writing-Original Draft, Writing-Review & Editing. XX: Software, Writing-Review & Editing. YA: Reviewing & Editing. LW: Supervision, Funding acquisition. KW: Conceptualization, Methodology, Writing-Review & Editing. All authors contributed to the article and approved the submitted version.

## Funding

This work was supported by the National Natural Science Foundation of China under Grant (No. 31702287); the Agricultural Science and Technology Innovation Program under Grant (talent training programs, No. 180818); and the earmarked fund for Modern Agroindustry Technology Research System from the Ministry of Agriculture of China under Grant (CARS-45).

## Conflict of Interest

The authors declare that the research was conducted in the absence of any commercial or financial relationships that could be construed as a potential conflict of interest.
